# Female Snub-Nosed Monkeys Exchange Grooming for Sex and Infant Handling

**DOI:** 10.1371/journal.pone.0074822

**Published:** 2013-09-25

**Authors:** Yang Yu, Zuo-Fu Xiang, Hui Yao, Cyril C. Grueter, Ming Li

**Affiliations:** 1 College of Life Science and Technology, Central South University of Forestry & Technology, Changsha, Hunan, China; 2 Key lab of Conservation Biology for Shennongjia Golden Monkey, Hubei Province, Shennongjia Forest District, Hubei, China; 3 School of Anatomy, Physiology and Human Biology, The University of Western Australia, Crawley, WA, Australia; 4 Key lab of Animal Ecology and Conservation Biology, Institute of Zoology, Chinese Academy of Sciences, Beijing, China; Université de Strasbourg, France

## Abstract

Allogrooming in primates has acquired an important social function beyond its original hygienic function and can be exchanged either for itself or used as a currency to obtain other benefits such as copulations, access to infants or agonistic support. We explore the strategic use of grooming as a social tool in semi-wild golden snub-nosed monkeys (*Rhinopithecus roxellana*) in central China, a species where two desirable resources, viz. reproductive males and infants, are restricted to the mating and birth season, respectively. We predict that females expend their grooming selectively to different individuals according to their “value”. Our results show that in the mating season, females devoted more grooming to the resident male than in the birth season, and this effect was particularly strong in non-mothers (females without newborn infants). Moreover, females were more likely to groom the resident male after copulation than during baseline social conditions. In the birth season, females devoted more grooming to other females than in the mating season, and mothers (females with newborn infants) were the most valuable grooming partners. The mean rate of contact by non-mothers toward infants of other females was significantly higher after grooming the mothers than in baseline social conditions. In conclusion, our findings lend credence to the notion that primate females use grooming as a strategic tool to obtain limited resources such as males and infants and vary preference for particular individuals depending on the seasonal availability of valuable resources.

## Introduction

Allogrooming (grooming hereafter) is probably the most common affiliative behavior among non-human primates and may serve multiple functions [1]. Grooming has likely evolved originally for a hygienic function [2]–[3], and then has been coopted for derived social purposes [4], including tension reduction [5]–[6], tolerance around resources [7], alliance formation and dominance acquisition [8]–[9], and group cohesion [10]. Grooming can also be seen as a strategic social tool or currency used to purchase return grooming from a partner [11]–[15] or a different commodity including agonistic support [9], [16]–[18] access to mates [19]–[21] and infants [22]–[24]. Primate affiliation, including allogrooming, can occur both between relatives and non-relatives [25]; while altruism among relatives is usually attributed to kin selection [26]–[27], altruism involving non-relatives can be a form of reciprocal altruism which assumes that the altruist later receives a significant benefit from the recipient of the initial altruistic act [28].

In non-human primates that breed year-round, desired resources such as infants and ‘reproductive males’ can become available at any time of the year, depending on individual female reproductive state. In seasonally breeding primates, these resources are available only during a short time window and are thus limited and should elicit competition. Competition over access to males is expected to be further exacerbated in social units containing only a single adult male [29]. Competition over access to infants is expected on the grounds that infants are extremely attractive [30]–[31], and handlers can gain maternal experience that will improve their ability to raise their own offspring as adults [32]. Seasonal fluctuations in supply of valuable resources are expected to produce asymmetries in efforts to acquire those resources (i.e. how frequently do females groom the male vs. other females).

Golden snub-nosed monkeys (*Rhinopithecus roxellana*) of central China exemplify a species in which both males and newborn infants represent seasonally limited resources for females. These primates live in a multi-level social system with two basic social units, one-male multi-female units (OMUs), i.e. bisexual reproductive units, and all-male units (AMUs), i.e. unisexual pre-or postreproductive units [33]–[35]. OMUs are held together by a network of male-female and female-female social interactions of varying strength, but the relative importance of same-sex vs. cross-sex relationships is debated [36]–[38]. Breeding of golden snub-nosed monkeys is strictly seasonal. Females conceive in autumn and give birth in spring [39]. If females give birth in the spring of one year, they will usually not become pregnant in the autumn of the same year, except in case of death of the newborn infant [40] Female golden snub-nosed monkeys are faced with multiple competitors in their OMUs and thus experience a high level of sexual competition in the mating season [29], [34], [41]. On the other hand, in the birth season, mothers with newborn infants are very attractive to other females. Females approach the mother, gather around her, groom the mother and the infant and try to get access to the infant [42].

A previous investigation into grooming reciprocity in a group of golden snub-nosed monkeys in Zhouzhi National Nature Reserve, northwest China, showed that the durations of grooming bouts offered and returned were asymmetrical between males and females. Males received more grooming from females than vice versa, and this pattern was stronger in OMUs with more females. Males received more grooming from females in the mating season than in the non-mating season, and female-to-male grooming time was correlated with copulation rate during the mating season [43]. In addition, Wei et al.’s [43] study also showed that females without infants preferentially groomed females with infants, and duration of grooming bouts given by non-mothers to mothers was negatively related to the number of infants present.

Based on the theoretical considerations outlined above, we predict that female golden snub-nosed monkeys use grooming strategically and allocate different amounts of grooming time to the resident male (‘grooming for mating’) and other females (‘grooming for inant handling’) in line with the seasonal availability of valuable resources. Specifically, we predicted the following: (1) females groom the resident male more often in the mating season than in the birth season, and in the mating season, non-mothers groom the resident male more often than mothers; (2) the rate of female-to-male grooming is higher after copulations than during baseline social conditions; (3) females groom other females more often in the birth season than in the mating season, and non-mothers groom mothers more often than other non-mothers. In addition, the mean rate of contact with infants of other females is higher after grooming the mothers than during baseline social conditions.

## Methods

### Ethics Statement

Permission to conduct this study was obtained from the Shennongjia National Nature Reserve and the State Forestry Administration of China, and all research protocols abide by the laws of the People’s Republic of China. Although the field studies involved endangered or protected species, we have got the approval from the state and local government. We recorded naturally occurring behavior in a non-invasive manner without engaging and/or interacting with the monkeys in any manner.

### Study Site and Subjects

The study was conducted at Dalongtan (2100 m *asl*) in Shennongjia National Nature Reserve (SNNR), Hubei, central China, in a mixed deciduous broadleaf/coniferous forest. At Dalongtan there is a group of golden snub-nosed monkeys that was habituated for tourism in 2006 and has been provisioned three to four times a day by reserve staff members ever since [44]. Provisioning might have influenced the monkeys’ social behavior and daily activities, especially in the early periods. However, they soon became habituated to the provisioning site, and instead of fleeing, the monkeys started ignoring the presence of researchers and reserve staff most of the time. As a result of successful habituation, observations could be made on a daily basis at distances between 3 and 50 m. At night, the monkeys’ roosts are typically located within a radius of 500 m from the provisioning site.

At the time of the present study, the study group (band) comprised 67 members divided into four OMUs named by the leader males (DD, XX, BT, DW) and one AMU containing three adult males. All of the adult individuals and most of the juveniles of both sexes were individually identifiable by unique physical features, such as body size, pelage color, body disability, the shapes of females’ nipples and the shapes of males’ granulomatous flanges on both sides of the upper lip. We chose two OMUs, units DD and XX, as focal units. DD unit included five adult females and two infants. XX unit included six adult females and three infants. For each female in these units, we recorded whether or not there was a newborn infant in that year. Copulations in the band were unevenly distributed across the year and a peak of sexual activity was recorded around October; birth occurred in the spring (March to May) (Xiang et al. in prep).

### Data Collection

Observations were conducted from August to November 2010 (71 days total), which was defined as the mating season, and from March to May 2011 (57 days total), defined as the birth season. Ten-minute focal animal sampling and continuous recording [45] were employed to record all grooming bouts involving the focal individuals, along with the identity of the participants. A grooming bout was considered to have ended when either the direction of grooming changed or when there was a break of more than 30 s. We collected focal samples of females via a randomized method, and for each female, we attempted to obtain an equal amount of observation time in each period during the study. If visual contact was lost with the focal subject, we abandoned these samples (Table 1).

**Table 1:** Observation time and details of grooming events for each female in the 2010 mating season and 2011 birth season.

Unit	ID	2010 mating season					2011 birth season				
		Birth(Y/N)^a^	Observationhours	Sample(N)	Grooming events to		Birth(Y/N)	Obs.Hours	Sample(N)	Grooming events to	
					Male^b^	Female^c^				Male	Female
DD	JJ	Y	24.83	149	13 (25%)	39 (75%)	N	14.83	89	8 (11%)	65 (89%)
DD	LN	N	26.17	157	29 (48%)	31 (52%)	Y	15.00	90	4 (11%)	32 (89%)
DD	YY	N	25.33	152	21 (49%)	22 (51%)	Y	14.83	89	6 (14%)	38 (86%)
DD	XL	Y	23.50	141	30 (33%)	62 (67%)	N	15.83	95	8 (15%)	47 (85%)
DD	GG	N	23.17	139	32 (52%)	30 (48%)	N	15.50	93	16 (25%)	48 (75%)
XX	NN	N	24.16	145	31 (38%)	51 (62%)	Y	13.17	79	8 (11%)	63 (89%)
XX	LL	N	24.00	144	26 (55%)	21 (45%)	Y	14.00	84	4 (10%)	37 (90%)
XX	BB	Y	24.83	149	23 (38%)	38 (62%)	N	14.83	89	11 (17%)	52 (83%)
XX	TT	Y	23.00	138	17 (21%)	63 (79%)	N	14.00	84	10 (16%)	51 (84%)
XX	WF	Y	23.17	139	20 (34%)	39 (66%)	N	15.83	95	16 (15%)	90 (85%)
XX	HHE	N	25.37	152	39 (42%)	53 (58%)	Y	15.00	90	5 (15%)	29 (85%)
	Total		267.50	1605	281	449		162.80	977	96	552

a Birth: whether females gave birth to an infant in the year, Y = Yes, N = No;

b Grooming events to the single resident male and the proportion (in parentheses) of all grooming events given by the female;

c Grooming events to other females and the proportion (in parentheses) of all grooming events given by the female.

To investigate whether a mating event increases the rate at which a female initiates grooming of the resident male we collected post-copulation (PC) samples. To investigate whether females are more likely to be allowed to handle infants after grooming their mothers, we collected post-grooming (PG) samples. PC samples were 10-min focal samples taken on a female that had just copulated with the resident male. PG samples were 10-min focal samples taken on a mother that had just received grooming. During PC samples, we recorded all grooming events between the focal female and the resident male. During PG samples, we scored all touches, grooming, and pick-ups of the focal mother’s infant [23], [46]. On the next possible day, at the same time, a 10-min matched-control observation (MC) was conducted which was matched with a PC or a PG sample [46]. In order to control for inflation of socio-positive interactions during PG and PC samples, we collected MC samples only if the two participants were in proximity (within 5 meters). If the two participants were not come into proximity during the MC samples and/or were involved in sexual or grooming interactions within two min preceding a planned MC, or in the first two min of an ongoing MC, the MC was postponed until the next possible day.

### Data Analysis

We analyzed only those grooming events that were free from conflicts to avoid potential biases, because grooming is also an expression of post-conflict affiliation or reconciliation [47]–[49].

An independent samples *t*-test was used to test for differences in females’ grooming rates (events per 10-min sample, calculated per day) directed both to the resident male and to other females between the mating and birth season. We also used an independent sample *t*-test to establish whether mothers and non-mothers differed in terms of grooming rates directed to the resident male in the mating season. A paired sample *t*-test was used to compare rates of grooming that non-mothers directed to mothers vs. other non-mothers in the birth season. A paired sample *t*-test was also used to compare grooming and infant contact rates between focal samples and PC/PG samples, and between PC/PG samples and MC samples.

Given that we found no significant differences in grooming and infant contact rates between the two OMUs, we combined the data for the two OMUs and present overall data and statistics. We used SPSS 19.0 statistical package to analyze the data. We set α = 0.05. Tests were always 2-tailed.

## Results

We observed a total of 1,378 grooming events initiated by females, including 377 events directed to the resident male and 1,001 events directed to other females (Table 1).

**Figure 1:**
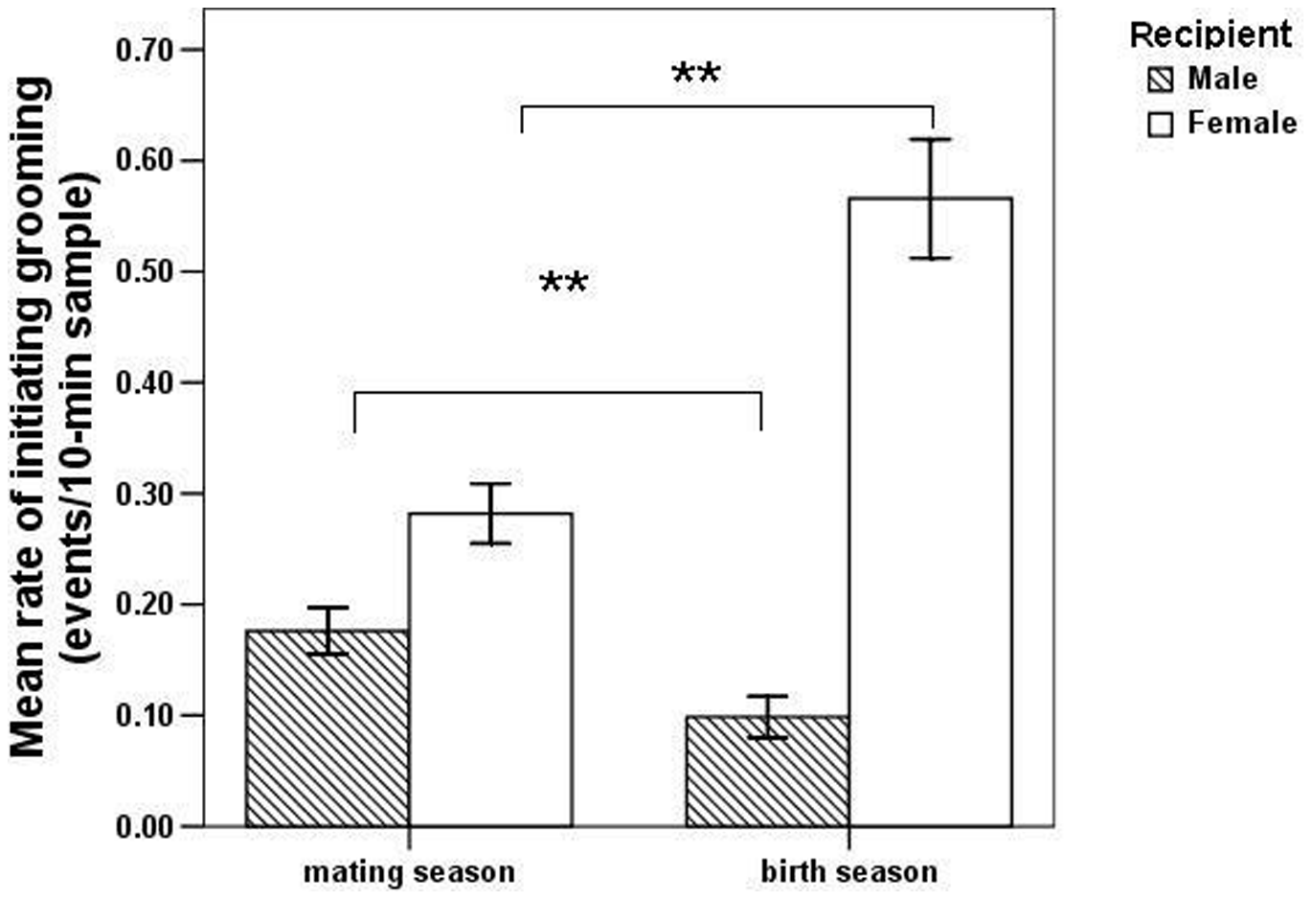
Mean rate (± SE) of female-to-male and female-to-female grooming compared between the mating and birth season (**indicates *p*<0.01).

**Figure 2:**
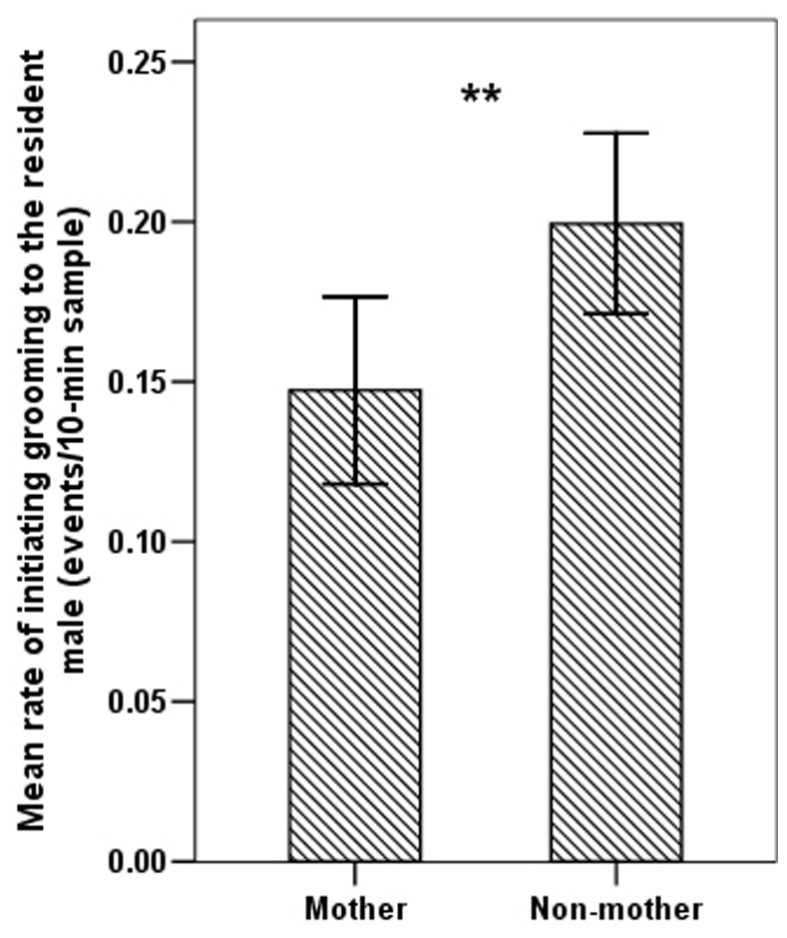
Mean rate (± SE) of grooming directed towards the resident male by mothers and non-mothers (**indicates p<0.01).

**Figure 3:**
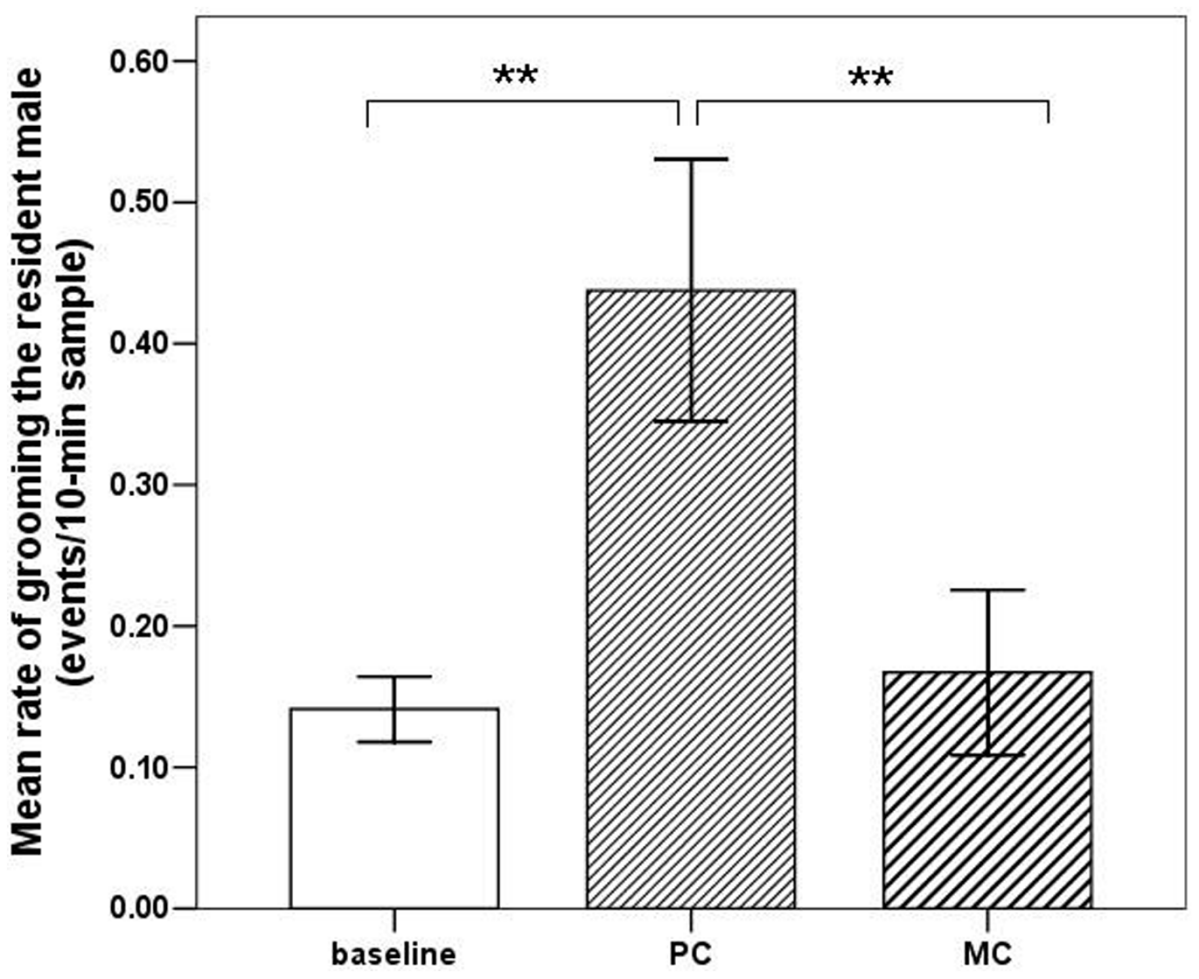
Mean rate (± SE) at which females directed grooming towards the resident male. The baseline bar represents the mean from focal sample data. The PC bar represents the mean from post-copulation sample data. The MC bar represents the mean from MC sample data where females and the single resident male were in proximity (**indicates *p*<0.01).

The mean rate of grooming directed by females to the resident male was significantly higher in the mating season than the birth season (independent samples *t*-test: *t* = 5.575, *df* = 134.975, *p*<0.001; Fig. 1). In the mating season, non-mothers showed significantly higher rates of grooming of the resident male than of mothers (independent samples *t*-test: *t* = −2.571, *df = *76, *p = *0.012; Fig. 2). The mean rate of grooming the resident male was significantly higher in PC samples than in focal samples (paired sample *t*-test *t* = −6.583, *df* = 10, *p*<0.001; Fig. 3). The PC and MC grooming rates were also significantly different (paired sample *t*-test *t* = 8.056, *df* = 10, *p*<0.001; Fig. 3). Female-to-male grooming rate was higher in PC than in MC samples (Fig. 3).

**Figure 4:**
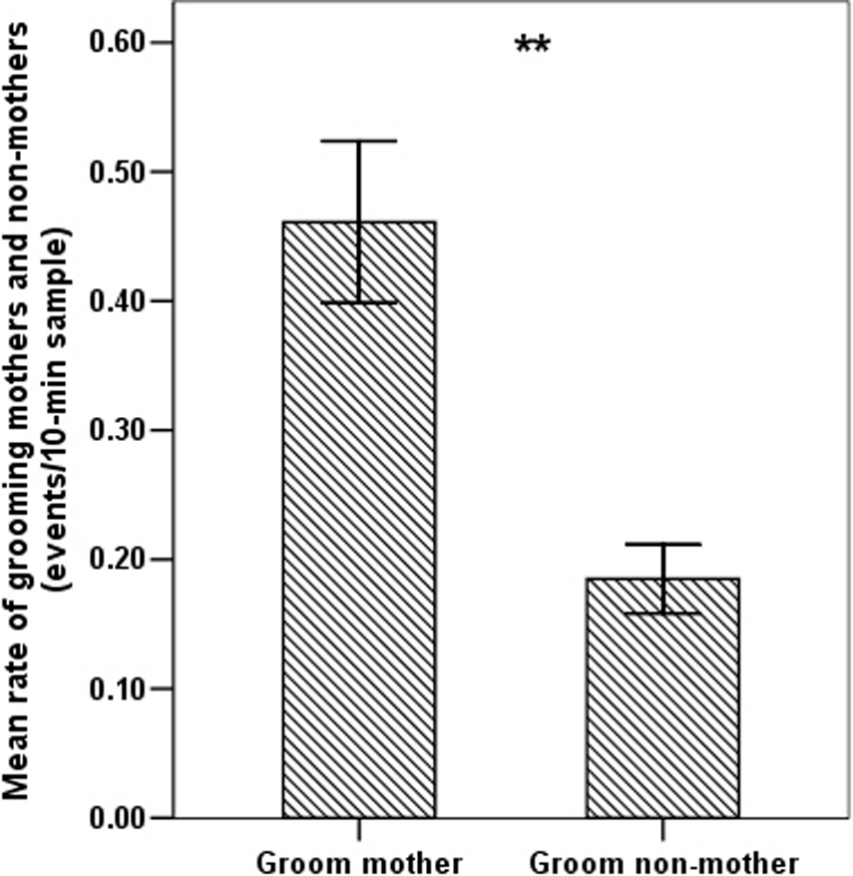
Mean rate (± SE) at which females without infants directed grooming towards mothers and non-mothers (**indicates *p*<0.01).

**Figure 5:**
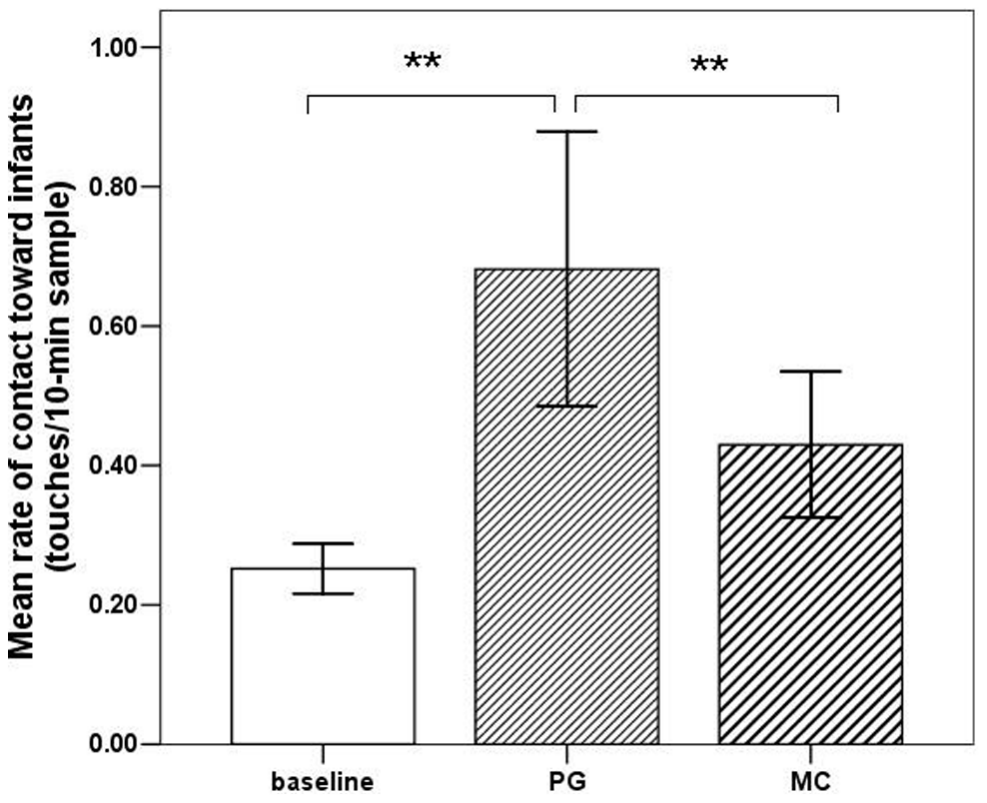
Mean rate (± SE) at which females established contact with infants. The baseline bar represents the mean from focal sample data. The PG bar represents the mean from post-grooming sample data. The MC bar represents the mean from MC sample data where mothers and non-mothers were in proximity (**indicates *p*<0.01).

Females groomed other females at significantly higher rates in the birth season than in the mating season (independent sample *t*-test: *t* = −9.470, *df = *87.489, *p*<0.001; Fig. 1). In the birth season, non-mothers devoted more grooming to mothers compared with other non-mothers (to mothers: 0.46 events/10-min sample; to non-mothers: 0.18 events/10-min sample; paired sample *t*-test: *t = *7.768, *df* = 32, *p*<0.001; Fig. 4). During baseline social conditions (focal samples), the mean rate of contact by females toward infants staying with their mothers was 0.25 touches per 10-min sample. After female-to-mother grooming (PG samples), the mean rate of contact toward infants with their mothers was 0.68 touches per 10-min sample, i.e. significantly higher than the baseline rate (paired sample *t*-test: *t* = 6.852, *df* = 4, *p* = 0.002; Fig. 5). In MC samples, the mean rate of contact toward the infant by the same female from the PG samples was 0.43 touches per 10-min sample, i.e. significantly lower than the PG rate (paired sample *t*-test: *t* = 7.328, *df* = 4, *p* = 0.002; Fig. 5).

## Discussion

This study supports the idea that female golden snub-nosed monkeys direct grooming effort strategically to those individuals that are most valuable to them in particular seasons. That is, females groomed the single resident male more frequently in the mating season than in the birth season, whereas females groomed other females more frequently in the birth season than in the mating season. There could be two reasons for this sex-specific allocation of grooming time shown by females. First, female-female competition over access to the single resident male, who is usually the exclusive mating partner in each OMU, is elevated during the mating season [29], [50]. Females would therefore be expected to devote more grooming to the single resident male in order to secure more mating opportunities. Another reason is that female golden snub-nosed monkeys, like virtually all Asian colobine females [51], are intensely interested in other females’ newborn infants and are highly motivated to interact with them [42]. Since newborn infants represent a limited resource for females in the birth season, prospective allocaretakers have to devote more grooming to mothers in order to increase their probability of being allowed to access and contact an infant.

Wei et al’s study [43] demonstrated that female golden snub-nosed monkeys in another population devoted more time to grooming their male during the mating season than during the non-mating season and non-mothers were attracted to mothers and directed more grooming towards them. Our results are consistent with these findings. The same study also found a positive correlation between copulation rate and time invested by females in grooming their male. In addition, when infants were in short supply, the duration of grooming bouts from non-mothers to mothers lasted longer. These findings indicated that golden snub-nosed monkeys focused their grooming on seasonally valuable partners and exchanged grooming with other sociosexual behaviors. We also investigated the temporal relationship between grooming bouts and copulatory events as well as incidents of allocare and detected reciprocation of services over a short timescale.

In another study on long-tailed macaques (*Macaca fasicularis*), males exchanged grooming for copulation opportunities [20]. Most male-to-female grooming occurred when females were receptive, and when sexual activity (e.g. mating, mounting, genital inspection, female presentation of the hindquarters) was involved, males groomed females longer. This was seen as evidence that grooming might be used as a payment for copulation, but only by males. Long-tailed macaque males used grooming to obtain immediate access to sexual resources, but females did not. What we found in our study group of golden snub-nosed monkeys was exactly the opposite. Our results show that females groomed the resident male more often after copulations than during baseline social conditions. The reason for this might be that as a polygynous harem-forming species, male golden snub-nosed monkeys are a limited resource for females which face multiple same-sex competitors and experience high levels of intrasexual competition for the attention of males [29], [52]. Generally speaking, our results indicate that female golden snub-nosed monkeys use grooming in return for copulations.

Our results also show that non-mothers groomed mothers more often than other non-mothers in the birth season and the rate of contact toward newborn infants from non-mothers was higher after they groomed a mother than during baseline social conditions. The PG-MC analysis also indicates that the mean rate of contact toward infants was higher after a female groomed a mother than during a matched control condition in which the female and the mother were just in proximity without contacts. These results point to the existence of an exchange system in which females trade grooming for infant handling. Previous studies on chacma baboons (*Papio cynocephalus ursinus*) [22], long-tailed macaques (*M. fascicularis*) [23] and olive baboons (*Papio anubis*) [53] provided evidence for the existence of such an infant market. In these species, grooming was mostly given by handlers to mothers and exchanged for infant handling. Henzi and Barrett [22] found that female chacma baboons without infants groomed mothers to gain access to their infants. Similar findings for female long-tailed macaques support the conclusion that grooming is used as a payment for access to infants [22]. Frank and Silk [53] also found partial support for the infant market hypothesis, i.e. female olive baboons offered more grooming to mothers than non-mothers and groomed mothers longer when they handled their infants than when they did not. Our data are in accord with these findings and support the conclusion that grooming and infant-handling are traded in golden snub-nosed monkeys.

In conclusion, our results suggest that female golden snub-nosed monkeys allocate their grooming time in a strategic fashion to obtain resources and services that are seasonally restricted, i.e. copulations and access to newborns. An increased focus on grooming the single resident male in the mating season results in better mating opportunities for females. Grooming can also be regarded as a payment by females to the male after mating. Grooming directed at females with newborns in the birth season facilitates infant handling by non-mothers, thus supporting the prediction that these two behaviors are exchanged.
